# Paeoniflorin adjunct to scar release surgery for postoperative anorectal stenosis: a randomized controlled trial

**DOI:** 10.3389/fsurg.2026.1843542

**Published:** 2026-09-03

**Authors:** Yuening Feng, Xi Wang, Dayong Feng, Chunhui Wang, Zhiyong Bai, Jingwen Wang, Yuhang Shen, Ayue An

**Affiliations:** 1 Wangjing Hospital of China Academy of Chinese Medical Sciences, Beijing, China; 2 The 305 Hospital of PLA, Beijing, China

**Keywords:** anorectal stenosis, antifibrotic therapy, defecation function, fibrosis, randomized controlled trial, SCAR, Shaobei injection, traditional Chinese medicine

## Abstract

**Background:**

Postoperative anorectal stenosis is a disabling complication of anorectal surgery, characterized by fibrotic narrowing that impairs defecation and quality of life. While scar-release surgery can restore lumen patency, long-term outcomes are undermined by recurrent fibrosis. Paeoniflorin, the active component of Shaobei Injection, is a promising botanical antifibrotic agent that may enhance perioperative efficacy. This study aims to evaluate whether combining Paeoniflorin with longitudinal scar-release surgery improves anatomical and functional outcomes in patients with postoperative anorectal stenosis compared to surgery alone.

**Methods:**

In this prospective, multicenter randomized controlled trial (Trial registration: ChiCTR2200062631) in China. Eligible patients with iatrogenic fibrotic anorectal stenosis were randomized equally (1:1) to receive either scar-release surgery plus Paeoniflorin (experimental group) or surgery alone (control group). The primary endpoints were stricture relief rate (defined as anal canal diameter ≥20 mm) and improvement in defecation difficulty score at 1 and 6 months. Secondary outcomes included anal pain (VAS), scar tissue thickness (imaging), and stricture recurrence rate. All patients were followed for 6 months.

**Results:**

A total of 128 patients were enrolled and randomized (64 per group); after excluding 22 patients lost to follow-up, 106 patients (53 per group) completed the 6-month follow-up and were included in the final analysis. At 6 months, the stricture relief rate was significantly higher in the experimental group compared to the control group (90.6% vs. 73.6%; *P* < 0.05; RR, 1.23; 95%CI, 1.03–1.48). Mean defecation difficulty scores improved significantly in both groups, but the experimental group maintained near-normal scores (0.70 ± 0.27 at 1 month and 0.78 ± 0.17 at 6 months) compared to the control group (2.23 ± 0.88 and 2.34 ± 0.51, respectively; *P* < 0.05 for both time points). Secondary outcomes including reduced scar thickness and lower recurrence rates (3.8% vs. 13.2%, *P* = 0.08; RR, 0.29; 95%CI, 0.06–1.31) further favored the combined therapy. No injection-related adverse effects were observed.

**Conclusions:**

These preliminary 6-month findings suggest that local paeoniflorin may provide exploratory benefits as an adjunct to scar-release surgery. Larger, longer-term trials are required to validate its clinical efficacy and durability.

**Clinical Trial Registration:**

https://www.chictr.org.cn/, ChiCTR2200062631.

## Introduction

Rectal or anal canal stenosis is an infrequent but disabling sequel of anorectal surgery. Modern series put the cumulative incidence at 0.2–7.5% after stapled haemorrhoidopexy (PPH). A recent retrospective study of 75 PPH cases reported stenosis in 6.7% of patients (1), while classical stapled rectal mucosectomy cohorts document a crude rate of 3.1% (2). After conventional open hemorrhoidectomy, the risk is lower but still clinically relevant, ranging from 1.5% to 3.8% and rising sharply when excessive anoderm is excised or wound infection supervenes (3). Once established, the rigid fibrotic ring reduces luminal compliance; patients develop constipation, painful or incomplete defecation and may enter a vicious cycle of trauma-induced scar amplification that markedly erodes quality of life (4).

Management is tiered according to severity. Clinic-based controlled manual anal dilatation offers prompt relief, yet long-term data show recurrence-free survival falls to 69% by five years (5), repeat procedures may be required in some patients, with a minority developing mild fecal incontinence on long-term follow-up (6). For fixed fibrotic rings, surgeons favour longitudinal or radial scar release or various advancement-flap anoplasties. A 2022 systematic review of 556 cases demonstrated that, despite pooled success rates exceeding 85%, anoplasty for anatomical anal stenosis still carries an overall morbidity burden, with complication rates of 10.2-11.5% and recurrence rates of 4.7% at ≥12 months of follow-up (7); other comparative series echo these limitations, particularly in extensive strictures (8). To optimize management strategies, contemporary clinical practice increasingly relies on advanced imaging modalities to evaluate pelvic floor dynamics and anorectal outlet obstruction. Techniques such as dynamic pelvic floor imaging, high-resolution transrectal ultrasonography, and magnetic resonance (MR) defecography play a pivotal role in distinguishing structural abnormalities from functional defecatory disorders (9). These modalities demonstrate that structural changes, including localized tissue hypertrophy, increased muscular thickness, and fibrotic scar formation, can impair the coordinated biomechanics of the anorectal junction during defecation. Accordingly, comprehensive clinical assessment should integrate objective anatomical parameters, such as scar thickness, with patient-reported functional outcomes to provide a more complete evaluation of disease severity and treatment response.

The central problem is biological, not mechanical: TGF-β-Smad signaling plays a central biological role in tissue fibrosis, where injury or inflammation can rekindle a fibroproliferative response marked by exuberant collagen I/III deposition (10). Targeting that pathway pharmacologically is therefore an attractive adjunct. Paeoniflorin serves as the key bioactive component in An's Shaobei Injection (NMPA No. Z20030126), a Chinese patent medicine used as a sclerosing and anti-inflammatory agent in anorectal disorders. In a prospective cohort of 1,520 patients, it achieved a 97.5% three-year clinical efficacy rate for grade I-III internal haemorrhoids, with a significantly lower incidence of post-procedural anal stricture compared with a conventional injection therapy (11). Experimental work shows paeoniflorin suppresses TGF-β-mediated epithelial-mesenchymal transition via Smad-dependent up-regulation of the inhibitory Smad7 (10), supporting an anti-fibrotic rationale. A preliminary clinical observation from our centres suggested that peri-lesional paeoniflorin delivered at the time of scar-release surgery improved luminal calibre and pain scores beyond surgery alone (12).

Against this background, within the framework of Traditional Medicine, we conducted an exploratory randomized controlled trial to investigate the preliminary clinical efficacy and safety of paeoniflorin (the primary bioactive component of Shaobei injection) combined with longitudinal scar incision versus scar release surgery alone for postoperative anorectal stenosis, focusing on stricture resolution, defecatory function, and scar remodeling.

## Methods

### Study design and patients

This is a randomized controlled trial conducting at two hospitals in China (ChiCTR2200062631). Patients with iatrogenic fibrotic anorectal stenosis (due to prior anorectal surgeries) were enrolled and randomly assigned (1:1) to an experimental group (scar-release surgery plus paeoniflorin) or a control group (scar-release surgery alone). Key inclusion criteria were: (1) presence of an anal/rectal stricture ring within 8 cm of the anal verge following procedures like stapled hemorrhoidopexy (PPH), circumferential hemorrhoidectomy, or sclerosant injection, with endoscopic evidence of purely iatrogenic fibrotic stricture; (2) symptoms of difficult defecation and examination showing a narrowed anal canal; and (3) consent to undergo the trial treatment and follow-up. To ensure a homogeneous study population, patients with non-iatrogenic anorectal strictures (including those of congenital origin, associated with inflammatory bowel disease, or secondary to pelvic radiotherapy), neurogenic bowel dysfunction, or acute anorectal conditions (e.g., anorectal abscess) were excluded. All patients provided informed consent.

### Treatments

The surgical intervention for all patients was longitudinal incision and transverse expansion scar lysis, a procedure in which fibrotic scar tissue at the stenosis is longitudinally incised (at typically 3, 6, and 9 o'clock positions) under anesthesia, followed by gentle transverse stretching/dilation of the scar ring until the lumen is adequately enlarged. In the experimental group, after exposing the stricture ring, paeoniflorin (diluted 1:1 with 0.25% lidocaine, total 20 mL) was infiltrated into the base of the fibrotic scar ring circumferentially before performing the standard incisions. The control group received the same surgical scar release technique but without the paeoniflorin injection. All procedures were performed under appropriate anesthesia (local or regional) with an anoscope for visualization.

Postoperatively, all patients received identical care: starting the day after surgery, dressings were removed, and patients were allowed normal diet and ambulation. The anal canal was gently dilated and irrigated twice daily under an anoscope. A sterile latex tube wrapped in hypertonic saline gauze was placed in the anal canal at the stricture site for 3-5 days to maintain patency, after which it was replaced with a tube wrapped in a topical herbal ointment gauze (Kangfuxin solution) for continued stent effect. Intravenous antibiotics and analgesics were given for 5 days, and warm sitz baths with traditional medicated decoction were used after defecation, in order to reduce infection and encourage healing. No other anti-fibrotic medications were applied in the control group, to isolate the effect of paeoniflorin.

### Endpoints and assessments

The primary endpoints were (1) stricture relief rate at 1 month and 6 months after surgery, defined as the proportion of patients achieving a minimum rectal lumen diameter ≥20 mm on exam (indicating resolution of the stenosis), and (2) improvement in defecation function score, assessed by a standardized questionnaire. Defecation difficulty was scored (with higher scores indicating worse constipation/difficulty) based on stool form and ease of passage for three consecutive bowel movements (with patients on a normal diet and no laxatives), using a scoring system referencing the Bristol Stool Form Scale (each patient's baseline preoperative score was compared to their postoperative scores at 1 and 6 months). The scoring system defines four clinical severity grades as follows: 0 points: Smooth and unobstructed defecation, with soft-formed stools; 2 points: Mildly obstructed defecation, but stools can still be passed spontaneously; 4 points: Strenuous defecation with significantly prolonged evacuation times; 6 points: Severe difficulty in defecation, requiring clinical assistant measures to pass stools.

Secondary endpoints included: postoperative anal pain assessed by the Visual Analog Scale (VAS, 0 = no pain, 10 = worst pain) at multiple time points (preoperatively, postoperative day 1, day 7, 1 month, and 6 months); scar thickness at the stenosis site measured by imaging (transrectal ultrasound or CT) at 1 month and 6 months compared to preoperative baseline; and stricture recurrence rate within 6 months, defined clinically as return of defecation difficulty (straining or incomplete evacuation) accompanied by an inability to comfortably pass an index finger on digital rectal exam, requiring intervention (dilation or reoperation). All adverse events and complications were recorded.

Scar thickness at the stricture site was assessed using a standardized imaging protocol. Most patients underwent high-resolution transrectal ultrasonography, whereas pelvic computed tomography (CT) was performed in a small subset of patients (3 in the control group and 2 in the experimental group). All imaging studies and scar thickness measurements were independently evaluated by two experienced clinicians from the radiology and ultrasound departments, both of whom were blinded to treatment allocation and clinical information throughout the study. Before study initiation, the two assessors completed a standardized calibration process to harmonize measurement techniques and anatomical boundary definitions for anorectal scar tissue. Transrectal ultrasonography was performed using a 360° rotating endoprobe to assess the anatomical extent of anorectal strictures. Patients were placed in the left lateral decubitus position, and the probe was gently inserted into the rectal lumen. At the narrowest level of the stricture, fibrotic scar tissue was visualized as a distinct hypoechoic to mixed-echoic circumferential thickening adjacent to the subepithelial interface. Scar thickness was measured using electronic calipers oriented perpendicular to the longitudinal axis of the anal canal. The measurement was obtained at the thickest point of the identified scar band, extending from the innermost mucosal/submucosal border to the outer margin of the muscularis propria or the outer boundary of the surrounding scar tissue, as illustrated in Supplementary Figure 1. The maximum scar thickness was recorded as a single value to represent the greatest extent of fibrotic thickening associated with the postoperative stricture.

Measurement reproducibility was evaluated in a randomly selected subset of 30 patients using the intraclass correlation coefficient (ICC). The ICC was 0.88 (95% CI, 0.81–0.93) for interobserver agreement and 0.91 (95% CI, 0.85–0.95) for intraobserver reproducibility, indicating excellent measurement reliability. To minimize potential variability introduced by different imaging modalities, transrectal ultrasonography served as the primary imaging modality. A prespecified sensitivity analysis restricted to patients who underwent transrectal ultrasonography was performed to verify that inclusion of the small CT subgroup did not materially influence the anatomical findings.

### Sample size and randomization

The sample size was calculated based on an expected difference in stricture resolution rates between groups. From preliminary data, we anticipated an effective rate of 94.8% in the injection group vs. 72.6% in the surgery-alone group (12). Using a two-tailed test with *α*=0.05 and power (1-β) = 90%, we estimated that around 53 patients per group would be required to detect a significant difference. To account for an estimated 20% loss to follow-up or dropout, the target enrollment was set at 64 patients per group (128 total). Patients were randomized in equal allocation via a centralized randomization system (third-party) to ensure concealment; group assignments were generated and managed by one independent researcher, and surgeons were informed of assignment on the day of surgery.

### Statistical analysis

Data were analyzed using SPSS 22.0. Because 22 randomized participants were lost to follow-up and had no available post-randomization outcome data, efficacy analyses were conducted by per-protocol analysis. Continuous variables (e.g., defecation score, VAS pain, scar thickness) are presented as mean ± standard deviation. Between-group comparisons for continuous data were performed with the independent-samples *t* test if data were approximately normally distributed and with equal variances; otherwise, a non-parametric test (such as Mann–Whitney) was used. Within-group comparisons (e.g., change from baseline) used paired *t* tests for parametric data. Categorical data (e.g., stricture relief rate, recurrence rate) were compared using Chi-square or Fisher's exact test. All tests were two-sided, with *P* < 0.05 considered statistically significant.

## Results

### Patient characteristics

After screening 157 cases, a total of 128 patients with postoperative anorectal stricture were enrolled, with 64 patients assigned to the experimental group and 64 to the control group. Eleven patients were lost to follow-up in each group, and 106 patients were ultimately included in per-protocol analysis (Supplementary Figure 2). At baseline, all patients presented with significant fibrotic anal strictures (Table 1), characterized by a minimal anal canal diameter of less than 20 mm (per inclusion criteria). The distribution of stricture severity (mild, moderate, severe) was similar across both groups. Defecation difficulty scores, based on a validated symptom scale (higher scores indicating more severe difficulty), were comparable between the experimental (5.01 ± 0.47) and control (4.91 ± 0.69) groups (*P* > 0.05). Pain severity was also high and similar in both groups at baseline, with visual analog scale (VAS) scores averaging 7.12 ± 0.87 in the experimental group and 7.09 ± 0.74 in the control group (*P* > 0.05). Mean scar thickness at the stricture site, measured via imaging, was 4.38 ± 0.44 mm and 4.41 ± 0.39 mm in the experimental and control groups, respectively (*P* > 0.05).

**Table 1:** Baseline characteristics.

Characteristics	Experimental group (*n* = 64)	Control group (*n* = 64)	Overall (*n* = 128)	*P*-value
Age (years)	33.5 (26–67)	36 (30–68)	35 (26–68)	0.41
Sex				0.78
Male	31 (48.4%)	29 (45.3%)	60 (46.8%)	
Female	33 (51.6%)	35 (54.7%)	68 (53.1%)	
Severity of Stenosis				0.69
Mild	38 (59.3%)	36 (56.3%)	74 (57.8%)	
Moderate	20 (30.2%)	23 (35.9%)	43 (33.6%)	
Severe	6 (9.4%)	5 (7.8%)	11 (8.6%)	
Defecation Difficulty Score	5.01 ± 0.47	4.91 ± 0.69		0.56
Pain Score (VAS)	7.12 ± 0.87	7.09 ± 0.74		0.88
Scar Thickness (mm)	4.38 ± 0.44	4.41 ± 0.39		0.80

### Stricture relief and defecation function

Of the 128 enrolled, 106 patients (53 in each group) completed the full 6-month follow-up. The remaining 22 patients were lost to follow-up and excluded due to incomplete data. All patients experienced immediate anatomical improvement in anal caliber after surgery. At postoperative Day 1, 100% (53/53) of patients in both groups achieved relief of the stricture (anal canal patent ≥20 mm on exam) due to the surgical incisions (Table 2). This remained 100% in both groups at Day 7, as the surgical sites were still dilated. However, differences emerged with longer follow-up. By 1 month postoperatively, the paeoniflorin group maintained a higher stricture relief rate (90.6%) compared to 81.1% in the surgery-only control group. By 6 months, 90.6% of the injection group still had a resolved stricture, versus only 73.6% of controls. The between-group differences at 6 months were statistically significant (*P* < 0.05; RR, 1.23; 95%CI, 1.03–1.48). These results suggest that the addition of paeoniflorin led to a higher long-term success rate in relieving the stenosis. Table 2 summarizes the stricture relief rates over time.

**Table 2:** Composite time-course of clinical and functional outcomes between the two groups.

	Stricture relief rates (%)			Defecation difficulty scores			Anal pain scores (VAS)		
Time point	Experimental	Control	*P*	Experimental	Control	*P*	Experimental	Control	*P*
Pre-treatment	–	–	–	5.01 ± 0.47	4.91 ± 0.69	0.56	7.12 ± 0.87	7.09 ± 0.74	0.88
Postop Day 1	53/53 (100%)	53/53 (100%)	1.000	0.80 ± 0.76	1.43 ± 0.99	>0.05	5.70 ± 0.70	5.95 ± 0.63	0.10
Postop Day 7	53/53 (100%)	53/53 (100%)	1.000	0.90 ± 0.78	2.06 ± 0.92	>0.05	4.20 ± 0.63	4.20 ± 0.68	0.99
Postop 1 Month	48/53 (90.6%)	43/53 (81.1%)	0.164	0.70 ± 0.27	2.23 ± 0.88	<0.05^b^	2.20 ± 0.39	3.75 ± 0.59	<0.001^d^
Postop 6 Months	48/53 (90.6%)	39/53 (73.6%)	<0.05^a^	0.78 ± 0.17	2.34 ± 0.51	<0.05^c^	1.20 ± 0.39	3.00 ± 0.65	<0.01^e^

a Risk Ratio was 1.23 (95%CI, 1.03–1.48).

b mean difference was −1.53 (95%CI, −1.78 to −1.28).

c mean difference was −1.56 (95%CI, −1.70 to −1.42).

d mean difference was −1.55 (95%CI, −1.74 to −1.36).

e mean difference was −1.80 (95%CI, −2.03 to −1.57).

Pre-treatment defecation scores (on a difficulty scale) were high in both groups (around 5 out of a maximum indicating severe difficulty) with no difference between groups. After intervention, both groups showed dramatic improvements in bowel function, but the experimental group improved more and sustained the improvement. On postoperative Day 1, the average defecation difficulty score dropped to 0.80 ± 0.76 in the injection group and 1.43 ± 0.99 in the control group. This day 1 difference was not statistically significant (*P* > 0.05), indicating that initially both groups experienced substantial relief of obstruction with the surgery. At 1 month, the injection group obtained near-normal defecation with an average score 0.70 ± 0.27, whereas the control group's difficulty score worsened to 2.23 ± 0.88; this difference was significant (*P* < 0.05; MD = −1.53; 95%CI, −1.78 to −1.28). The trend persisted at 6 months: experimental group 0.78 ± 0.17 vs. control 2.34 ± 0.51, (*P* < 0.05; MD = −1.56; 95%CI, −1.70 to −1.42). Within each group, defecation scores improved markedly from pre-surgery levels (by Day 1 both groups had *P* < 0.05 vs. baseline, and remained improved at all later times), but the magnitude of improvement was greater in the injection group in the long term. Table 2 presents the defecation function scores over time. Clinically, by 6 months the majority of patients in the injection group reported normal or near-normal bowel movements, whereas a substantial fraction of control patients had return of straining, incomplete evacuation, or required continued self-dilation to pass stool.

These functional findings correspond with the objective stricture measurements, indicating that paeoniflorin provides a meaningful improvement in defecation quality and patient comfort in the long term.

### Postoperative pain

Post-surgical anal pain was assessed using VAS. Both groups experienced postoperative pain due to the surgical incisions, peaking on day 1 and gradually subsiding. Baseline pain levels (pre-surgery, due to the chronic fissure/stricture) were moderate-severe in both groups with no difference (Table 2). On Day 1 post-op, pain scores averaged 5.70 ± 0.70 in the injection group vs. 5.95 ± 0.63 in controls. This slight reduction in the experimental group was not statistically significant (*P* > 0.05) on day 1, indicating both groups had comparable immediate postoperative pain. By Day 7, pain had decreased in both groups with no significant difference between groups at that time (*P* > 0.05).

However, at later follow-ups, the injection group reported significantly less pain than controls. At 1 month, the mean VAS in the experimental group was 2.20 ± 0.39, compared to 3.75 ± 0.59 in the control group (*P* < 0.001; MD = −1.55; 95%CI, −1.74 to −1.36) (Table 2). At 6 months, the injection group's pain was nearly negligible (1.20 ± 0.39), while the control group had a mean VAS of 3.00 ± 0.65, indicating some persistent discomfort; this difference was significant (*P* < 0.01; MD = −1.80; 95%CI, −2.03 to −1.57) (Table 2). These results suggest that paeoniflorin contributed to a greater reduction in long-term anal pain, potentially by promoting better healing and reducing chronic inflammation or fibrosis that can cause pain. These differences correlate with the differences observed in scar tissue and stricture recurrence, indicating better long-term comfort with the combined therapy.

### Scar tissue thickness

Imaging assessments (via anal ultrasound and/or CT) were used to measure the thickness of scar tissue at the stricture site, as an objective indicator of fibrosis. Before treatment, the average scar thickness was 4.38 ± 0.44 mm and 4.41 ± 0.39 mm in the experimental and control groups, respectively, with no difference between the two groups (Table 3). At 1-month post-op, both groups showed a reduction in measured scar thickness due to the surgical scar incisions and initial remodeling: the experimental group's average scar thickness was 2.80 ± 0.38 mm and the control group's was 3.46 ± 0.75 mm. Although the injection group's scar appeared thinner on average, the difference at 1 month was not statistically significant (*P* > 0.05), likely due to variability and the relatively short time for scar remodeling.

**Table 3:** Scar thickness at stricture site (imaging measurement, mm).

Time point	Experimental (injection)	Control (no injection)	*P* value
All Patients (Pooled, *n* = 106)			
Pre-treatment, mm	4.38 ± 0.44	4.41 ± 0.39	0.80
1 Month Post-op, mm	2.80 ± 0.38	3.46 ± 0.75	0.09
6 Months Post-op, mm	0.90 ± 0.28	3.06 ± 0.36	<0.05^a^
Ultrasound Subgroup (*n* = 101)			
Pre-treatment, mm	4.36 ± 0.45	4.40 ± 0.40	0.82
1 Month Post-op, mm	2.77 ± 0.36	3.45 ± 0.73	0.09
6 Months Post-op, mm	0.88 ± 0.23	3.04 ± 0.35	<0.05

a mean difference was −2.16 (95%CI, −2.28 to −2.04) mm.

By 6 months, however, a clear divergence was evident: the paeoniflorin group had a mean scar thickness of only 0.90 ± 0.28 mm, indicating a very thin, flexible scar at the treated site, whereas the control group had a mean scar thickness of 3.06 ± 0.36 mm, reflecting persistent fibrotic tissue. This difference was highly significant (*P* < 0.05; MD = −2.16; 95%CI, −2.28 to −2.04 mm). Thus, at 6 months the injection-treated patients essentially had minimal residual scar, whereas controls had a scar about three times thicker on average. These imaging findings support that paeoniflorin markedly inhibits scar tissue regeneration and promotes scar resolution over time. To minimize potential bias arising from the use of different imaging modalities, a subgroup analysis was performed including only patients who underwent transrectal ultrasonography (*n* = 51 in the experimental group and *n* = 50 in the control group). The findings were consistent with those of the overall cohort, with no significant between-group difference in preoperative scar thickness (4.36 ± 0.45 mm vs. 4.40 ± 0.40 mm, *P* = 0.82), a nonsignificant trend toward a thinner scar at 1 month postoperatively (2.77 ± 0.36 mm vs. 3.45 ± 0.73 mm, *P* = 0.09), and a significantly thinner scar at 6 months postoperatively (0.88 ± 0.23 mm vs. 3.04 ± 0.35 mm, *P* < 0.05). These findings support the robustness of the observed treatment effect regardless of the imaging modality used. Table 3 presents the scar thickness comparisons.

These objective measures of fibrosis correspond well with the clinical outcomes: patients with thinner scars had wider lumens and fewer symptoms. The lack of significant difference at 1 month suggests that by that time the scarring process was still ongoing; the striking difference at 6 months demonstrates the long-term antifibrotic effect of the injection. Figure-based evaluations of the scar also indicated better restoration of normal tissue architecture in the experimental group, whereas controls often showed hypertrophic scar formation on imaging at 6 months.

### Recurrence rate

**Figure 1:**
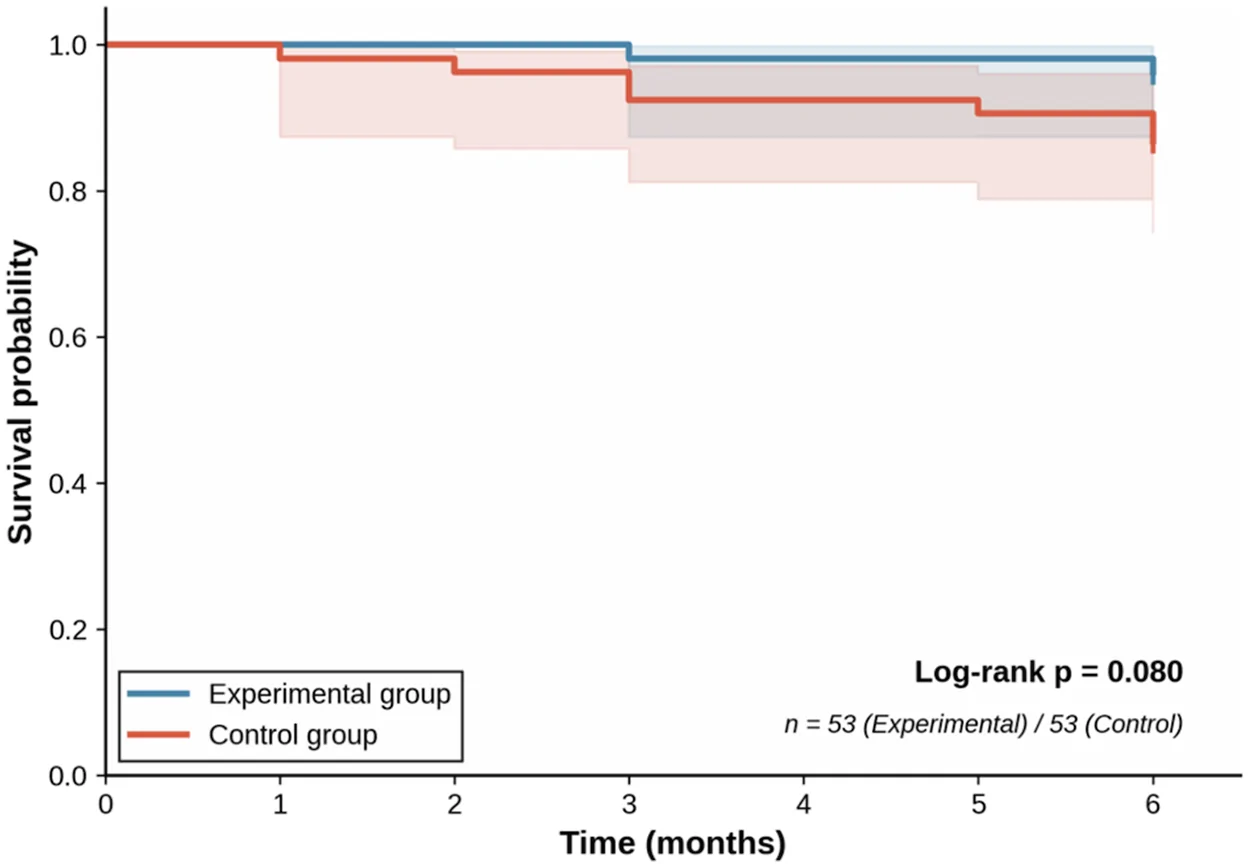
Kaplan–Meier survival curves for recurrence-free survival. Kaplan–Meier estimates of recurrence-free survival are shown for the experimental group (*n* = 53) and the control group (*n* = 53) over a 6-month follow-up period. The survival curves demonstrate the probability of remaining free from recurrence over time. Censored observations are indicated by vertical tick marks on the survival curves. The number of patients at risk at each time point is presented below the *x*-axis. The difference between the two groups was assessed using the log-rank test (*p* = 0.080). Shaded areas represent 95% confidence intervals.

By the 6-month follow-up, we assessed if patients had experienced a clinical recurrence of the stricture, defined by return of difficult defecation and the need for further intervention. In the paeoniflorin group, only 2 patients (3.8%) had evidence of stricture recurrence within 6 months. In contrast, 7 patients (13.2%) in the control group had recurrence of significant stenosis and symptoms by 6 months. This difference in recurrence rates was marginal significant (*P* = 0.08). Kaplan–Meier analysis further demonstrated that the experimental group had a higher recurrence-free survival rate than the control group (96.2% vs. 86.8%; log-rank test, *P* = 0.08), with the survival curves diverging over time (Figure 1). The combination therapy appeared to reduce the recurrence of anal stenosis at 6 months. A summary is provided in Table 4.

**Table 4:** Six-month stricture recurrence.

Group	Recurrence at 6 months (*n*, %)
Experimental (Injection)	2/53 (3.8%)
Control (No Injection)	7/53 (13.2%)
Risk ratio	0.29 (95%CI, 0.06–1.31)
*P* value	0.08

### Safety outcomes

Comprehensive surgical-related complications were monitored throughout the follow-up period. In the experimental group, no instances of postoperative bleeding or infection were observed. In the control group, two patients (3.8%) developed localized infection at 14 days postoperatively, characterized primarily by anal tenesmus and increased wound secretions; both cases resolved completely following a 5-day course of targeted anti-infective therapy. No episodes of severe hemorrhage, urinary retention, or other major procedure-related adverse events were recorded in either group. These findings confirm that the addition of paeoniflorin did not introduce additional safety risks and was associated with an overall favorable tolerability profile.

## Discussion

This exploratory study suggests that paeoniflorin combined with scar-incision release surgery may offer potential benefits over surgery alone for postoperative anorectal stenosis, warranting further investigation. The combined therapy led to higher stricture resolution rates and sustained patency of the rectal lumen, translating into better functional outcomes (improved defecation with less need for straining) and reduced long-term pain. The objective imaging findings were also directionally consistent with the observed clinical recurrence pattern. At 6 months, the experimental group demonstrated significantly thinner scar tissue and a numerically lower recurrence rate than the control group (3.8% vs. 13.2%), indirectly suggesting a potential association between reduced scar formation and a lower risk of symptomatic restenosis. Beyond the statistical differences observed in anal canal caliber and sonographic scar thickness, these findings hold meaningful practical clinical relevance. The notable reduction in the need for secondary reinterventions (such as repeat dilations or reoperations) combined with sustained improvements in defecatory symptoms underscores the potential real-world benefit of local paeoniflorin administration. Although the magnitude of difference in certain secondary parameters may appear modest, preventing symptomatic recurrence and sparing patients from additional invasive procedures represent a major pragmatic advantage of this combined approach.

Scar reformation leading to recurrent stenosis is a well-known issue after these procedures, with success rates often in the range of 70-80% at 6-12 months, depending on the severity and patient factors (13). In our control group (surgery alone), we observed a 73.6% stricture relief rate at 6 months, which is consistent with prior reports of such surgical outcomes. Prior work on anal stricture treatments has mostly focused on surgical techniques and on adjuvant measures like local flaps or dilators (7). The concept of injecting an anti-scar agent is somewhat analogous to the use of intralesional steroids for strictures or keloids, which have shown efficacy in some reports. Our results are in concordance with those reports - confirming that locally targeting fibrosis can indeed improve outcomes of mechanical stricture release. In the context of anorectal surgery, Chinese practitioners have reported anecdotal success using various medicinal ointments or injections to improve wound healing; however, rigorous data were lacking (14).

The observed higher long-term patency rate in the injection group (91% vs. 74%) suggests a potentially favorable trend. Furthermore, patients experienced better functional results - an aspect sometimes overlooked when focusing on anatomical success. The defecation score used in our study, while not a standard international score, effectively captured the patient's ease of bowel movements. The injection group's scores remained near 0, whereas the control group increased over time, indicating a relapse of symptoms. This illustrates that beyond anatomic measurements, the patients’ quality of life was markedly better with the addition of paeoniflorin. We also observed a significant difference in pain at 1 and 6 months. Chronic anal pain in stenosis often comes from recurrent fissuring or mucosal ulceration on the tight scar band. By preventing the scar band from reforming tightly, the injection likely spared patients from these painful complications, explaining the lower VAS scores in the long run.

These functional improvements highlight the clinical importance of integrating precise anatomical evaluations with advanced functional imaging in the modern assessment of evacuation disorders. In clinical practice, differentiating between structural mechanical obstruction (such as the fixed postoperative stenosis addressed in this study) and functional defecatory disorders (such as dyssynergic defecation) remains a diagnostic challenge. Recent work has increasingly emphasized the indispensable value of dynamic pelvic floor imaging, particularly magnetic resonance defecography and dynamic ultrasound, in distinguishing functional from structural causes of obstructed defecation. In patients presenting with manometric dyssynergia, for instance, a recently published study by Ölçücüoğlu et al. demonstrated that MR defecography provides pivotal diagnostic value by identifying coexisting or mimicking structural evacuation disorders, thereby preventing inappropriate functional therapies and guiding targeted structural interventions (9).

Furthermore, the relationship between objective anatomical imaging parameters and functional defecatory outcomes is well-supported by previous investigations into imaging-related biomarkers. For example, Çamur and Acar investigated morphometric markers such as puborectalis muscle and abdominal subcutaneous adipose tissue thickness on imaging, demonstrating how specific structural and anatomical parameters correlate with, and potentially indicate, underlying dyssynergic defecation (15). In our trial, the significant correlation between the imaging-derived reduction in scar thickness and the clinical resolution of defecation difficulty aligns with this paradigm. By utilizing high-resolution transrectal ultrasound to monitor scar regression, we could objectively document that reducing structural tissue hypertrophy directly translates into restored defecatory biomechanics. This reinforces the growing consensus that advanced radiological and sonographic parameters are not merely secondary endpoints, but essential markers that contextualize and predict functional outcomes in complex anorectal disorders.

Given that inflammation and TGF-β-mediated fibrosis are key drivers of scar formation (16), adjunct therapies that target these processes could improve results. Corticosteroid injection into strictures has been used in other contexts (e.g., esophageal or bowel strictures) to inhibit collagen deposition; in fact, intralesional steroid injections alongside dilations have been shown to prolong the patency of benign GI strictures and delay recurrence (17). However, steroid use carries risks (e.g., tissue atrophy, infection) and may not be widely accepted for anal stenosis. Unlike intralesional steroids, paeoniflorin exerts its anti-fibrotic effect via TGF-β/Smad modulation without causing local tissue damage or increased susceptibility to infection. In our series, no cases of atrophy or injection-related infection occurred in the experimental group, whereas two control patients developed transient wound infections.

Paeoniflorin, the principal bioactive component derived from traditional herbal formulations (such as An's Shaobei Injection, NMPA Z20030126), has demonstrated potent anti-inflammatory and anti-fibrotic effects in anorectal conditions. Clinical studies in hemorrhoid therapy have shown that this therapy can safely reduce edema, bleeding, and promote tissue remodeling (11, 18, 19). Its mechanism is progressively being elucidated; preclinical evidence demonstrates that paeoniflorin inhibits TGF-β/Smad signaling by upregulating the inhibitory mediator Smad7, thereby attenuating fibroblast activation and extracellular matrix deposition (10, 20, 21). While this preclinical work provides a plausible theoretical framework for our clinical findings of reduced scar thickness and improved luminal patency, it is critical to note that direct molecular and histopathological evidence was not captured within the framework of this trial. Therefore, these clinical benefits should be viewed as exploratory findings within an integrative medicine approach.

The addition of paeoniflorin to scar-release surgery is a relatively simple intervention that can be easily adopted. It adds minimal time to the procedure and did not result in any notable adverse effects in our series. The safety profile was excellent: no injection-site complications were seen. This is important given that some anti-fibrotic agents (like steroids or chemotherapy drugs) could theoretically impair wound healing or cause infection (22, 23); we saw no such issues - in fact, all patients healed well, and injection patients if anything healed with a more supple scar. The findings suggest that surgeons dealing with anal stricture - particularly those due to postoperative scarring may consider incorporating an antifibrotic injection at the time of scar incision. Paeoniflorin is currently available in China; for international practice where this specific product might not be accessible, our study opens the door for exploring similar strategies (for example, other anti-fibrotic injectables or perhaps platelet-rich plasma, etc., though those need their own evidence). The principle demonstrated is that targeting the scar formation process can significantly enhance the efficacy of mechanical stricture treatments. This combined approach could reduce the need for more aggressive surgeries like full-thickness anoplasty or colostomy, and spare patients from repeated interventions.

The criterion of an anal luminal diameter ≥20 mm was selected as a pragmatic indicator of successful stricture relief rather than a universally validated anatomical cutoff. Although no consensus threshold has been established because of individual anatomical variation, previous surgical studies have similarly adopted calibrated restoration of the anal lumen as a marker of successful reconstruction. For example, Meter et al. (24) targeted a postoperative anal caliber of 25–26 mm during calibrated diamond flap anoplasty, whereas Farid et al. (25) reported favorable functional outcomes with postoperative anal calibers of approximately 20–24 mm following reconstructive procedures for anal stenosis. These findings support the clinical relevance of our predefined endpoint, although future studies integrating anatomical measurements with patient-reported functional outcomes are warranted to establish a standardized definition of treatment success.

We acknowledge several limitations of this study. First, the follow-up duration of 6 months, while sufficient to assess short-term recurrence, is relatively short for a chronic issue like fibrosis. Strictures could potentially recur after 6 months; ongoing follow-up of these patients is needed to see if the benefits of injection persist at 1 year or beyond. Second, our outcome assessment for defecation difficulty was based on a novel scoring method (tailored to our setting); while it was derived from a recognized scale (Bristol stool form and defecation symptoms), it has not been externally validated. That said, the large between-group differences and consistency with other measures support its reliability in this context. Third, this trial was conducted in specialized centers in China, the generalizability of our results was limited. Fourth, the efficacy analyses were based on per-protocol analysis. Although loss to follow-up was balanced between the two groups and remained within the attrition rate anticipated in the sample-size calculation, the exclusion of participants without post-randomization outcome data may have introduced attrition bias and should be considered when interpreting the findings. Fifth, the absence of direct biological evidence, including histopathological, molecular, and biomarker assessments, limits mechanistic interpretation. Although the clinical and imaging findings are consistent with an antifibrotic effect, the underlying mechanisms cannot be directly confirmed. Sixth, given the limited 6-month follow-up and regional scope, these findings should be interpreted as exploratory and situated within the conceptual framework of Traditional Chinese Medicine integrative therapy. Larger, multi-center trials with standardized global formulations are needed to validate these outcomes.

In conclusion, within the framework of Traditional Medicine, these findings provide exploratory evidence that local paeoniflorin administration—as the primary bioactive component of Shaobei injection—may serve as a potential adjunct to longitudinal scar-release surgery for postoperative anorectal stenosis. The observed trends in stricture relief, functional improvement, and reduced scar thickness suggest that targeting local fibrotic responses with botanical compounds warrants further investigation. Nevertheless, given the relatively short 6-month follow-up, modest sample size, and localized clinical context, these results must be interpreted as exploratory rather than definitive. Long-term, large-scale randomized controlled trials with extended follow-up and mechanistic biomarker analyses are required to confirm the therapeutic durability, optimal dosing, and broader applicability of paeoniflorin in surgical patient management.

## Data Availability

The raw data supporting the conclusions of this article will be made available by the authors, without undue reservation.
