# Supplementary material for: Paeoniflorin adjunct to scar release surgery for postoperative anorectal stenosis: a randomized controlled trial
Source: Front Surg. 2026 Sep 3;13:1843542. doi: 10.3389/fsurg.2026.1843542 (PMC13582448; doi:10.3389/fsurg.2026.1843542)
Supplement: Supplementary file 1 [file Supplementaryfile1.docx]

**Supplementary materials**

**Supplementary Figure 1. Representative transrectal ultrasound imaging demonstrating the scar thickness measurement methodology at baseline.**

The 360-degree endosonic scan at the level of the post-surgical stenotic ring visualizes the circumferential cicatricial tissue. The electronic calipers (yellow '+' markers) measure the radial thickness of the hypoechoic fibrotic scar band perpendicular to the luminal interface at its thickest point. In this representative preoperative case, the maximum scar thickness is measured at 0.424 cm, demonstrating significant tissue hypertrophy restricting the anorectal canal.

**Supplementary figure 2. Inclusion flowchart**
